# Using ^15^N-Ammonium to Characterise and Map Potassium Binding Sites in Proteins by NMR Spectroscopy

**DOI:** 10.1002/cbic.201300700

**Published:** 2014-02-12

**Authors:** Nicolas D Werbeck, John Kirkpatrick, Jochen Reinstein, D Flemming Hansen

**Affiliations:** [a] Institute of Structural and Molecular Biology, Division of Biosciences, University College London Gower Street, London, WC1E 6BT (UK); [b] Department of Biomolecular Mechanisms, Max Planck Institute for Medical Research Jahnstrasse 29 69120 Heidelberg (Germany)

**Keywords:** ammonium, enzyme catalysis, HDAC, Hsp70, NMR spectroscopy, potassium binding

## Abstract

A variety of enzymes are activated by the binding of potassium ions. The potassium binding sites of these enzymes are very specific, but ammonium ions can often replace potassium ions in vitro because of their similar ionic radii. In these cases, ammonium can be used as a proxy for potassium to characterise potassium binding sites in enzymes: the ^1^H,^15^N spin-pair of enzyme-bound ^15^NH_4_^+^ can be probed by ^15^N-edited heteronuclear NMR experiments. Here, we demonstrate the use of NMR spectroscopy to characterise binding of ammonium ions to two different enzymes: human histone deacetylase 8 (HDAC8), which is activated allosterically by potassium, and the bacterial Hsp70 homologue DnaK, for which potassium is an integral part of the active site. Ammonium activates both enzymes in a similar way to potassium, thus supporting this non-invasive approach. Furthermore, we present an approach to map the observed binding site onto the structure of HDAC8. Our method for mapping the binding site is general and does not require chemical shift assignment of the enzyme resonances.

## Introduction

Many enzymes are activated by specific binding of one or more monovalent cations (MVCs); these either contribute directly to the catalysis at the active site or indirectly modulate the activity by binding to an allosteric site. Potassium and sodium ions are the most common activating MVCs, with a strong correlation between MVC preference and intracellular (K^+^) or extracellular (Na^+^) location of the enzyme.[1] Examples of intracellular, potassium-activated enzymes are the molecular chaperones Hsp70[2] and GroEL[3] (K^+^ binding at the active sites is crucial for the ATP-dependent reaction cycles), ribokinase (K^+^ binding near but not at the active site activates the enzyme),[4] and the hydrolase histone deacetylase 8 (HDAC8; binding to two K^+^ binding sites affects activity).[5] It is generally found that enzymes that are activated by the binding of K^+^ (ionic radius 1.33 Å) are either not activated or are activated to a lesser extent by Na^+^ (ionic radius 0.97 Å), and vice versa. However, enzymes that are activated by K^+^ can often be activated by an MVC of similar ionic radius, such as Rb^+^ (1.48 Å) or NH_4_^+^ (1.44 Å).[6] In some cases it has even been possible to engineer a potassium-independent enzyme by introducing a lysine residue in such a way that its terminal R-NH_3_^+^ occupies the binding site, thereby taking the place of the regulatory potassium ion.[7]

Using NH_4_^+^ as a proxy for K^+^ offers the opportunity to characterise potassium binding sites by NMR spectroscopy, as isotopically labelled ^15^NH_4_^+^ is readily available and can be employed in combination with conventional heteronuclear solution-state NMR experiments. The use of ^15^NH_4_^+^ as a K^+^ mimic has been reported previously for the characterisation of potassium binding sites of DNA quadruplexes[8] by NMR spectroscopy. However, the previously investigated DNA quadruplexes are relatively small compared to many enzymes, and quadruplexes also exhibit highly symmetric MVC binding sites. It is therefore not immediately obvious that the approach of using ^15^NH_4_^+^ to probe potassium binding sites will also be applicable to larger, potassium-dependent enzymes.

Herein we show that ^15^NH_4_^+^ can be used to probe K^+^ binding sites in medium-sized enzymes (>40 kDa) by NMR spectroscopy. The technique is facilitated by local reorientation and cross-correlated relaxations of the symmetric ^15^NH_4_^+^ molecule within its binding site; this results in line-narrowing of the ^15^N and ^1^H resonances, thereby improving the sensitivity of heteronuclear NMR experiments. In contrast to other experimental approaches used to probe MVC binding sites in proteins, NMR measurements of ^15^NH_4_^+^ have distinct advantages: 1) these probe the binding of the MVC directly, not indirectly (e.g., by enzyme activity or stability); 2) they have a high spatial resolution (in contrast to, for example, fluorescence spectroscopy); 3) they cause relatively little perturbation (unlike site-directed mutagenesis-based experiments); and 4) they are suitable for monitoring weak binding events (up to the high mm range), where other methods to study MVC binding (e.g., isothermal titration calorimetry) have limitations.[9]

The critical point is that the binding of ^15^NH_4_^+^ to an MVC binding site can decrease the exchange of the ammonium protons with the bulk solvent to such an extent that these become observable even under conditions where free ^15^NH_4_^+^ in solution is undetectable in proton–nitrogen-correlated experiments because of rapid exchange with the solvent.

For the HDAC8 enzyme, we first show that ammonium and potassium regulate the activity similarly, thus supporting the use of ^15^NH_4_^+^ as a proxy for K^+^. Using ^15^N-edited NMR methods, we probed the binding of ^15^NH_4_^+^ to one of the potassium binding sites of HDAC8 and to two potassium binding sites of the bacterial Hsp70 homologue DnaK. Furthermore, we developed a strategy for mapping potassium binding sites within protein structures that does not require a protein chemical shift assignment. The strategy relies on selective isotope labelling, and utilises through-space NOE measurements between ^15^NH_4_^+^ protons and residue-specific side-chain protons.

## Results and Discussion

### Binding of ammonium to HDAC8

**Figure 1:**
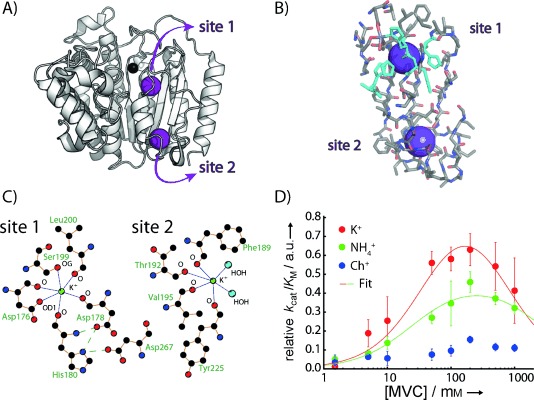
A) Crystal structure of HDAC8 (PDB ID: 2V5W) with the active-site Zn^2+^ (black sphere) and the two K^+^ ions (purple spheres). B) Residues within approximately 10 Å of the two potassium binding sites in HDAC8. Histidine residues are shown in cyan. C) Two-dimensional representations of the two binding sites (generated with Ligplot).[12] K^+^-coordinating residues and atoms are labelled. D) Activity profile as a function of monovalent cation concentration. Concentrations of K^+^ (red), NH_4_^+^ (green) and choline^+^ (blue) were in 25 mm Tris⋅HCl at pH 8.0. The two-site binding model described previously[5] was used to fit the activity profile for KCl and NH_4_Cl according to the equation *v*=*v*_1_/(1+*K*_1/2,act_/[MVC]+[MVC]/*K*_1/2,inh_), where *K*_1/2,act_ and *K*_1/2,inh_ are the apparent binding affinities (dissociation constants) for activation and inhibition, *v* is the relative *k*_cat_/*K*_M_ value, *v*_1_ is the *k*_cat_/*K*_M_ value of the fully activated state and [MVC] is the concentration of the respective monovalent cation. *k*_cat_/*K*_M_ of the fully inhibited state was set as zero; data are normalised such that *v*_1_ (K^+^)=1.0. *K*_1/2,act_ (K^+^): 45±26 mm, *K*_1/2,inh_ (K^+^): 610±530 mm, *K*_1/2,act_ (NH_4_^+^): 25±8 mm, *K*_1/2,inh_ (NH_4_^+^): 2600±1700 mm. Although the errors for the affinities of the inhibitory site are large, these data agree with a bell-shaped activity profile for K^+^ and NH_4_^+^.

The 42 kDa human HDAC8 enzyme is activated by the binding of potassium ions.[5] In addition to the catalytically important Zn^2+^ ion at the active site, crystal structures of HDAC8 reveal two MVC binding sites (Figure 1 A–C), as has been observed in most structurally characterised HDACs and HDAC-related deacetylases.[10] Biochemical characterisation[5] and computational approaches[11] have highlighted the importance of potassium binding for activity and potentially even for regulation of this enzyme. Specifically, the effect of KCl concentration on HDAC8 activity was investigated by Fierke and co-workers using HDAC8 with two different divalent metal ions (Co^2+^ and Zn^2+^) bound at the active site.[5] These experiments revealed a bell-shaped activity profile, with an activating effect at lower concentrations of K^+^ and an inactivating effect as the concentration was increased. The resulting activation (*K*_1/2,act_) and inhibition (*K*_1/2,inhib_) constants, which describe the apparent dissociation constants for an activating and an inhibiting binding site, respectively, were *K*_1/2,act_=14 mm and *K*_1/2,inhib_=130 mm for Zn^2+^-bound HDAC8.

In order to test whether NH_4_^+^ is a good mimic of K^+^ for enzyme activation, we measured the deacetylase activity of HDAC8 as a function of NH_4_^+^, K^+^ and choline concentration. Choline was used as a negative control to detect any possible nonspecific influence of ionic strength on HDAC8 activity. For K^+^ and NH_4_^+^ the activity profiles were bell-shaped (Figure 1 D), similar to those reported previously, whereas addition of choline had only a very small effect on HDAC8 activity. NH_4_^+^ activation of HDAC8 was ∼60 % of that of K^+^, with comparable *K*_1/2,act_ (compare the curves in Figure 1 D), thus suggesting that NH_4_^+^ effectively mimics K^+^ in modulating the activity of HDAC8.

**Figure 2:**
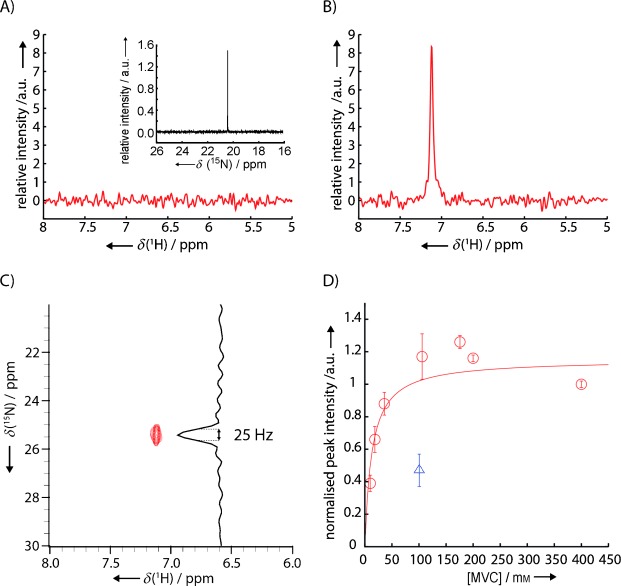
A) ^15^N-edited 1D proton spectrum of the buffer (25 mm Tris⋅HCl pH 8.0, 200 mm ^15^NH_4_Cl, 0.5 mm TCEP) and direct ^15^N-detected 1D spectrum (inset). B) As above but after addition of ∼100 μm [^14^N]HDAC8 to the sample. C) ^1^H,^15^N HSQC spectrum of the sample shown in B). D) Titration of ^15^NH_4_^+^ (○) with the observed binding site in HDAC8 (using ^15^N-edited 1D proton spectra). The intensities were obtained by integration of the ammonium peak, and errors were estimated by a combination of integrating multiple noise regions and by taking into account the uncertainties in calculating the relative protein concentration. At 100 mm ^15^NH_4_^+^, addition of 100 mm KCl (▵) led to approximately 50 % reduction of the signal, thus indicating that K^+^ and NH_4_^+^ bind to the same site with similar affinities. A hyperbolic binding equation *Y*=*A*×*X*/(*K*_D_+*X*) (—) was fitted to the normalised peak intensities to yield *K*_D_=(13±8) mm.

Next, we tested whether it was possible to observe binding of ^15^NH_4_^+^ to HDAC8 by NMR. As expected, bulk ^15^NH_4_^+^ was not observed in ^15^N-edited, proton-detected 1D NMR spectra in buffer containing 200 mm ^15^NH_4_^+^ at pH 8.0 (Figure 2 A) because of rapid exchange of the ammonium protons with the bulk solvent. The ^15^N chemical shift of the free ammonium was therefore determined by direct ^15^N-detection to be 20.5 ppm (Figure 2 A, inset). Addition of [^14^N]HDAC8, however, resulted in a distinct peak in the ^15^N-edited experiment, indicative of HDAC8-bound ^15^NH_4_^+^ with a proton chemical shift of 7.1 ppm (Figure 2 B). A ^15^N chemical shift of 25.5 ppm was subsequently determined by recording a 2D ^1^H,^15^N HSQC spectrum (Figure 2 C).

The ^15^N line-width of ^15^NH_4_^+^ at 298 K bound to HDAC8 (42 kDa) would be expected to be ∼45 Hz, with the ^15^N nucleus relaxed principally by dipolar interactions with its four protons, with a rotational correlation time of approximately 25 ns. The observation of a peak in the ^15^N-edited spectra, shifted from the position for free ammonium, shows that binding of the ^15^NH_4_^+^ ion to HDAC8 is sufficiently strong to significantly limit exchange of the ammonium protons with the bulk solvent. Although cross-correlated relaxations within the ^15^NH_4_^+^ molecule cause line-narrowing, the experimental line-width (∼25 Hz), which also includes contributions from the residual proton exchange and proton *R*_1_, indicates that the ammonium ion is undergoing local reorientation within the binding site. The spectral parameters obtained from the peak in the ^15^N-edited spectrum report directly on ^15^NH_4_^+^ in the MVC binding site, with the intensity of this peak directly related to the amount of ^15^NH_4_^+^ bound to HDAC8, thus allowing an estimation of the dissociation constant.

Proton-detected, ^15^N-edited 1D spectra were recorded for a titration of HDAC8 with ^15^NH_4_^+^, and the resulting ^15^NH_4_^+^ peak integrals were used to determine the dissociation constant for binding of ^15^NH_4_^+^ to HDAC8 (*K*_D_=(13±8) mm; Figure 2 D). Addition of 100 mm KCl to a sample containing 100 mm ^15^NH_4_^+^ led to a reduction (∼50 %) in the ^15^NH_4_^+^ integral, thus indicating both that NH_4_^+^ and K^+^ compete for the same binding site and that they bind to this site with similar affinities (Figure 2 D). We therefore conclude that NH_4_^+^ binds to at least one of the two K^+^ binding sites in HDAC8. Binding of NH_4_^+^ to this site decreased proton-exchange with the solvent to such an extent that it was observable in ^1^H,^15^N-correlated experiments, under conditions where bulk NH_4_^+^ cannot be detected. The change in ^15^N chemical shift upon binding (Δ*δ*=5 ppm) indicates a significant change in the chemical environment, and given the different natures of the two potassium binding sites in HDAC8 (see below and Figure 1 C), it is unlikely that both the proton and ^15^N chemical shifts of the two HDAC8-bound ammonium ions would be so similar as to lead to overlap of the corresponding peaks in the ^1^H,^15^N-correlated spectra. We propose instead that HDAC8 binds a second NH_4_^+^—as it binds a second K^+^—but that this second site does not protect the bound NH_4_^+^ from solvent proton exchange sufficiently to be detectable in our experiments.

### Binding of ammonium to DnaK

**Figure 3:**
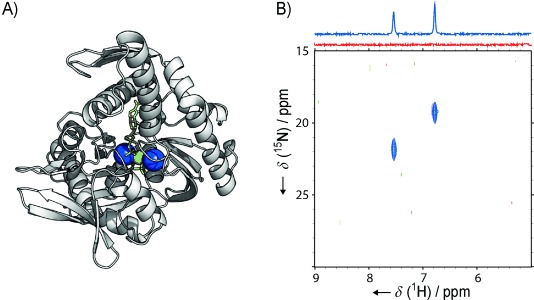
A) Crystal structure of the ATPase domain of Hsp70 (PDB ID: 1HPM):[2] purple spheres: K^+^, green sphere: Mg^2+^, yellow sticks: ADP and P_i_. A Ligplot analysis showing two-dimensional representations of the binding sites is shown in Figure S2. B) ^1^H,^15^N HSQC spectra and 1D projections (above) with the ABD of bacterial Hsp70 homologue DnaK. Red spectra: DnaK-ABD (100 μm) in 30 mm Tris⋅HCl, 150 mm ^15^NH_4_Cl, 5 mm MgCl_2_ and 1 mm DTT, pH 7.5. Blue spectra: the same sample after addition of 0.5 mm ADP and 50 mm ^14^NH_4_H_2_PO_4_. Note: because of the low pH of the ^14^NH_4_H_2_PO_4_ stock solution, Tris base was also added to achieve pH 7.5, thus increasing the total Tris concentration to 75 mm.

Another example of a potassium-binding enzyme is the molecular chaperone Hsp70. Two potassium ions in the ATP-binding domain (Figure 3 A) have been identified to be crucial for the ATPase cycle,[6b] which in turn regulates the binding and release of substrate proteins.[13] In a similar way to that for HDAC8, these potassium ions can be replaced by ammonium, thereby resulting in approximately half of the ATPase activity observed with potassium.[6b] Based on our success in characterising the potassium binding sites of HDAC8 with ^15^NH_4_^+^, we tested the general applicability of our approach by probing the potassium binding sites of the 41 kDa ATP-binding domain of DnaK, a bacterial Hsp70 homologue from *Thermus thermophilus*.

Unlike for the experiments with HDAC8, addition of nucleotide-free DnaK ATP-binding domain (DnaK-ABD) to a ^15^NH_4_Cl-containing buffer gave only a very weak signal in ^15^N-edited proton-detected and ^1^H,^15^N correlation spectra. The signal was barely above the noise level (Figure 3 B, Figure S1B in the Supporting Information), thus suggesting either that ^15^NH_4_^+^ binds only weakly to nucleotide-free DnaK-ABD, or that the binding sites are solvent-exposed and do not shield the ammonium protons from exchange with bulk solvent (Figure S1). Interestingly, addition of ADP and P_i_ resulted in the appearance of two clear peaks (Figure 3 B). The ^15^N chemical shifts of both these peaks (19.3 and 21.9 ppm) are different from the ^15^N chemical shift that we observed for free ammonium in this buffer when using a direct-detect ^15^N experiment (20.6 ppm). Our interpretation of these results is that binding of ADP and P_i_ protects the ammonium protons from solvent exchange, by themselves blocking solvent molecules and/or by inducing a conformational change that decreases solvent accessibility. Both effects agree well with molecular models of nucleotide binding to Hsp70, and it is likely that a combination of both leads to the increased protection of ammonium ions.[2, 14]

To verify that the two resonances observed in Figure 3 B report on two different sites, we recorded ^15^N-edited NOESY experiments (Figure S3). In the resulting ^15^N-edited NOESY spectrum, the two resonances gave rise to very different sets of cross-peaks, thus confirming that the resonances report on two different sites in the protein, in agreement with the observation of two different potassium binding sites in the crystal structures of Hsp70 chaperones.

### Mapping the MVC binding sites

The ammonium protons bound in a protein environment are close to protons of nearby amino acid side chains; this proximity serves as a potential means for mapping the MVC binding site within the protein structure. The key restraints used for protein structure determinations are nuclear Overhauser enhancements (NOEs) between protons, where the intensity of the observed NOE is approximately proportional to *r*^−6^ (*r* is the distance between the two interacting protons). Similarly, NOEs between bound ammonium protons and protein side-chain protons report on the location of the MVC binding site.

**Figure 4:**
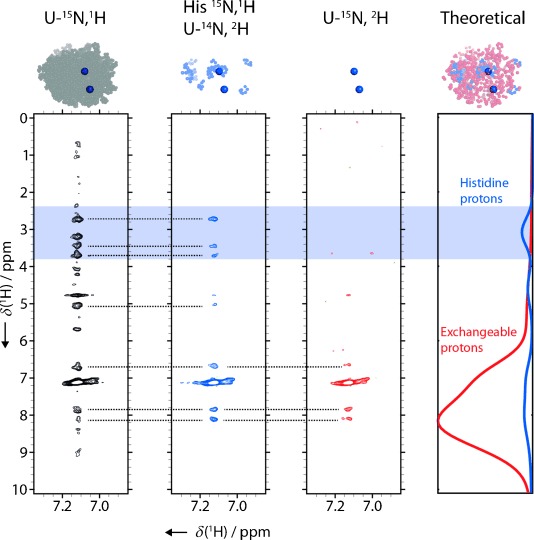
^15^N-edited NOESY experiments with different isotope labelling schemes used to map the K^+^/NH_4_^+^ binding site of HDAC8 (PDB ID: 2V5W[15]). Shaped ^15^N pulses at 25 ppm (see the Experimental Section) ensured selection of ammonium-specific cross-peaks. The fully protonated HDAC8 sample (left, black) gives rise to many cross-peaks that arise from the side chain and backbone protons of HDAC8 in the vicinity of the ammonium binding site. Expressing HDAC8 in a medium supplemented with protonated histidine in an otherwise deuterated background (middle, blue) reduces the number of crosspeaks significantly. The only observed cross-peaks in the aliphatic region are those from non-exchangeable histidine side-chain protons. A control experiment shows HDAC8 expressed in deuterated medium without labelled histidine supplement (right, red). Only solvent-exchangeable protons give rise to cross-peaks. The graph on the right shows the distributions of expected chemical shifts for the exchangeable protons in HDAC8 (red) and all histidine protons (blue) based on BMRB statistics. The HDAC8 structures above the spectra (modelled from PDB ID: 2V5W) highlight protons introduced by the corresponding labelling scheme in blue (histidine), red (exchangeable) and grey (all). This set of experiments, in combination with the distribution of histidine residues, localises the observed ammonium binding site in the HDAC8 structure.

A ^15^N-edited NOESY experiment was performed to identify side chains in the vicinity of the observed MVC binding site of HDAC8. The resulting spectrum shows a number of NOE crosspeaks between the HDAC8-bound ammonium and the aliphatic side chains, as well as between the ammonium and the amide protons (Figure 4). For proteins where a full chemical shift assignment is available, the binding site is readily mapped from the chemical shifts of the NOE cross-peaks. However, for HDAC8, as well as many other enzymes of interest, a full chemical shift assignment is not available, so alternative approaches must be used. Site-directed mutagenesis and mutation of K^+^-binding side chains might be an initial and intuitive approach. However, such an approach fails for HDAC8, as the potassium is mostly coordinated by the protein backbone, and also because mutation of side chains that do participate in the coordination of K^+^ (S199 and D176, see Figure 1 C)[15] resulted in insufficient purified protein for further NMR characterisation. Generally, identification of MVC binding sites by sitedirected mutagenesis could prove difficult, as such sites are either close to the active site or in allosteric regions of the enzyme. Site-directed mutagenesis therefore easily leads to large perturbations of the protein environment. We therefore prefer a non-invasive approach (described below) that is based on residue-specific isotope labelling.

Inspection of the two potassium binding sites in the HDAC8 crystal structure revealed different distributions of amino acids in the immediate vicinities of the two binding sites. Whereas the binding site close to the active site (here termed “site 1”) is surrounded by histidines (Figure 1 B and Figure 4) and leucines, these amino acids are not found in the vicinity of site 2. We therefore produced HDAC8 that was selectively protonated at histidine side chains, by adding ^1^H,^15^N-labelled histidine in an otherwise fully ^2^H,^14^N background. Histidine is at the end of a biosynthetic pathway and therefore little scrambling with other amino acids is expected.[16] This is confirmed by the fact that we observed a maximum of 11 cross-peaks in the amide region of a ^1^H,^15^N HSQC spectrum of this sample, compared to 13 histidines in HDAC8 (Figure S4). Although a specifically histidine-labelled sample has aliphatic protons only in the histidine side chains, additional protons are present at exchangeable sites, such as unprotected backbone amides. To account for exchangeable protons introduced by the H_2_O buffers, we expressed and purified a sample (U-^15^N,^2^H) in the same deuterated background and without the addition of protonated histidine. By comparing the results of the subsequent NOESY experiments (Figure 4), we could clearly identify several cross-peaks originating from histidine side chains. This demonstrates that the observed NH_4_^+^ binds to site 1, and thus we were able to assign the observed K^+^/NH_4_^+^ binding site in HDAC8 even in the absence of a full chemical shift assignment.

In general, identification and assignment of MVC binding sites is possible provided that 1) the corresponding ^15^NH_4_^+^ is sufficiently protected from solvent exchange that a signal is observed in ^15^N-edited NMR spectra, and 2) the MVC binding sites of interest have different local distributions of amino acids.

## Conclusions

We successfully used ^15^NH_4_^+^ to characterise a K^+^ binding site in the 42 kDa enzyme HDAC8 by NMR spectroscopy. By using ^15^NH_4_^+^ instead of K^+^, we could employ NOESY experiments in combination with selective amino acid labelling of the protein to probe the immediate vicinity of the binding site and map it onto the HDAC8 structure. These experiments have advantages over other means of characterising potassium binding sites in proteins. Firstly, these experiments map the potassium binding sites with minimal perturbation. As HDAC8 has two binding sites for potassium and these are connected by one β-strand, one would expect perturbations of these sites to have mutual effects on each other. Chemical shift perturbations upon titrating with K^+^ or assays based on site-directed mutagenesis could therefore lead to ambiguous results. The assignment of the sites by ^15^NH_4_^+^-detected NOESY spectra does not suffer from such ambiguity. Secondly, this strategy does not rely on an available chemical shift assignment for the protein, and is generally applicable provided a crystal structure is known and the amino acid compositions of the binding sites in question are different. And thirdly, most of the experiments presented here do not require isotopically labelled protein samples and are therefore relatively cost-effective compared to many other experiments involving NMR spectroscopy.

The bell-shaped activity profile and the presence of two MVC binding sites in crystal structures of HDAC8 led to a model of one activating and one deactivating K^+^ binding site. Combination of this model with site-directed mutagenesis experiments has led to the proposition that occupation of the K^+^ binding site close to the active site (site 1) is deactivating, whereas occupation of the second site (site 2) is activating.[5] Our results, however, suggest that site 1 is occupied even at relatively low concentrations of K^+^ (^15^NH_4_^+^ *K*_D_ ≈13 mm), that is, in the concentration range of enzyme activation. Recently, we were able to show that binding of potassium has an effect on the structural integrity of HDAC8 in a similar concentration range (K^+^ *K*_D_≈42 mm) as probed by an arginine side chain close to site 2 (∼12 Å from site 2, 21 Å from site 1).[17]

Based on these findings, we therefore propose that both potassium binding sites are important for the stabilisation and activation of the enzyme, and we speculate that the deactivating effect at higher potassium concentrations could result from secondary effects, such as that of high ionic strength on *K*_M_ or inhibition by high concentrations of chloride.[6a]

In a second example, we monitored ammonium binding to the 41 kDa ATP binding domain of the Hsp70 chaperone DnaK. After addition of ADP and P_i_, we observed two crosspeaks in the ^1^H,^15^N HSQC spectrum, in agreement with the two potassium ions observed in the crystal structure of Hsp70 with ADP and P_i_. These results are in agreement with previous findings of a closed and less solvent-accessible active site in response to nucleotide binding.[14a]

Overall, we detected three potassium binding sites in two proteins by using ^15^NH_4_Cl and ^15^N-edited NMR spectra. The observed chemical shifts range from 6.8 to 7.5 ppm in the ^1^H dimension and from 19 to 25.5 ppm in the ^15^N dimension. The level of dispersion suggests that this approach can be used to observe and identify multiple potassium binding sites in even large proteins, where the relaxation properties might be improved by cross-correlated relaxations and local reorientation of the ^15^NH_4_^+^ ion within the binding site.

The methodology presented above could be further exploited by combination with the powerful array of existing NMR techniques to describe the kinetic and dynamic aspects of MVC binding. For example, employing longitudinal exchange-type experiments would allow measurement of the exchange on the millisecond-to-second timescale between two or more bound MVCs, or the exchange of bound MVCs with those from the bulk solvent, as has been shown in the context of DNA quadruplex structures.[8b] The latter experiment depends on detection of bulk ammonium and thus requires a low pH (e.g., pH 5.0),[8b] which might prove a limitation for many proteins. It would also be intriguing to shed light on the relative mobility of ammonium ions in the binding sites of enzymes, and ^15^N,^1^H-relaxation-based experiments could give access to such information in the future. Given this potential, correlating characteristics of ^15^NH_4_^+^ binding with functional enzymatic states, as exemplified here, will ultimately lead to a better understanding of the role of monovalent cations in proteins and enzymes.

## Experimental Section

**Expression and purification of HDAC8:** Recombinant HDAC8 was expressed and purified as described previously.[17] Isotope-labelled samples were prepared from culture grown in M9 minimal medium. For the U-^15^N,^1^H sample, the M9 medium was made with ^15^NH_4_Cl (1 g L^−1^). For the U-^15^N,^2^H sample, the M9 medium was made up in ∼99 % D_2_O with ^15^NH_4_Cl (1 g L^−1^) and ^2^H_7_-glucose (2.5 g L^−1^). For the His-^15^N,^1^H/U-^14^N,^2^H sample, the M9 medium was made up in ∼99 % D_2_O with ^14^NH_4_Cl (1 g L^−1^) and ^2^H_7_-glucose (2.5 g L^−1^); [^15^N,^1^H]histidine (60 mg L^−1^) was added to the culture 1 h prior to induction. Expression was induced with ZnCl_2_ (0.2 mm) and isopropyl-β-d-thiogalactopyranoside (1 mm), and growth continued overnight at 21 °C before harvesting. Typical HDAC8 yields were 1–2 mg per litre of M9 culture. All isotopes were purchased from Sigma–Aldrich. Recombinant DnaK-ABD (residues 1–381 of *Thermus thermophilus* DnaK) was expressed in 2YT medium from BL21(DE3) cells and purified essentially as described previously for DnaK.[18] The yield was approximately 30 mg per litre of 2YT culture. The nucleotide content (∼2 %) of the preparation was determined by HPLC analysis.

**Activity assay:** HDAC8 activity was measured by a modified fluorescence-based assay.[19] The substrate analogue Boc-Lys(Ac)-7-amino-4-methylcoumarin (MAL) was synthesised according to Hoffmann et al.[20] and kindly provided by Christopher Matthews and Charles Marson (UCL, London). The deacetylation reaction was performed in Tris**⋅**HCl (25 mm, pH 8.0) with different concentrations of KCl, NH_4_Cl and choline chloride. HDAC8 (0.4 μm) was added to these buffers and incubated for at least 30 min at room temperature. It should be noted that this addition introduced KCl (final concentration 1.5 mm) from the enzyme stock. The reaction was started by adding substrate (200 μm), and quenched after 30 min at 25 °C by addition of an equal volume of developer solution (trypsin (10 mg mL^−1^) and trichostatin A (4 μm) in Tris**⋅**HCl (50 mm pH 8.0), NaCl (137 mm), KCl (2.7 mm), MgCl_2_ (1 mm), PEG (10 %, avg. 8000 Da) and BSA (1 mg mL^−1^)). After incubation at room temperature for 30 min, the degree of relative deacetylation was quantified in a FLUOstar Optima plate reader (excitation: 380 nm, detection: 460 nm; BMG Labtech, Aylesbury, UK).

**NMR experiments:** Unless indicated otherwise, samples for NMR experiments were prepared in Tris**⋅**HCl (25 mm, pH 8.0), with TCEP (0.5 mm), NaN_3_ (1 mm) and ^15^NH_4_Cl (200 mm). The spectra presented in Figure 2 and 3 were measured with an external D_2_O reference insert (Wilmad coaxial insert Z278513 (Sigma–Aldrich)), in which case no D_2_O was added to the sample buffer. Otherwise, NMR buffer contained 10 % D_2_O. Protein concentrations were 0.1–0.3 mm. All ^15^N-edited proton 1D spectra and the direct-observed ^15^N 1D spectra were recorded at 298 K on a 500 MHz Avance III spectrometer (Bruker) equipped with a room temperature, inverse, triple-resonance probe. The pulse sequence for the ^15^N-edited proton 1D spectra was based on the first increment of the fast ^15^N HSQC sequence.[21] The ^15^N carrier frequency was at 25 ppm. ^15^N-edited NOESY spectra were recorded at 298 K on a 700 MHz Avance III spectrometer (Bruker) equipped with a cryogenic inverse triple-resonance probe. The pulse sequence for the NOESY spectra was based on a standard NOESY/^15^N HSQC sequence,[22] with ammonium selectivity achieved by a shaped IBurp1 nitrogen pulse (1714 μs, centred at 25 ppm) in the first INEPT of the ^15^N HSQC element.[23] Broadband ^15^N decoupling during the indirect proton chemical shift evolution period was achieved by using an adiabatic smoothed CHIRP pulse (1000 μs, 30 kHz sweep-width, centred at 100 ppm).[24] The NOESY mixing time was 80 ms. Data were processed and visualised by using Topspin (Bruker), nmrDraw[25] and Sparky.[26]

## Supporting Information

As a service to our authors and readers, this journal provides supporting information supplied by the authors. Such materials are peer reviewed and may be re-organized for online delivery, but are not copy-edited or typeset. Technical support issues arising from supporting information (other than missing files) should be addressed to the authors.

miscellaneous_information
