# Supplementary material for: Using 15N-Ammonium to Characterise and Map Potassium Binding Sites in Proteins by NMR Spectroscopy
Source: Chembiochem. 2014 Feb 12;15(4):543–8. doi: 10.1002/cbic.201300700 (PMC4016748; doi:10.1002/cbic.201300700)
Supplement: Supplementary file 1 — miscellaneous_information [file cbic0015-0543-sd1.pdf]

CHEMBIOCHEM

Supporting Information

© Copyright Wiley-VCH Verlag GmbH & Co. KGaA, 69451 Weinheim, 2014

Using ^{15}N-Ammonium to Characterise and Map Potassium Binding Sites in Proteins by NMR Spectroscopy

Nicolas D. Werbeck,*[a] John Kirkpatrick,[a] Jochen Reinstein,[b] and D. Flemming Hansen*[a]

cbic_201300700_sm_miscellaneous_information.pdf

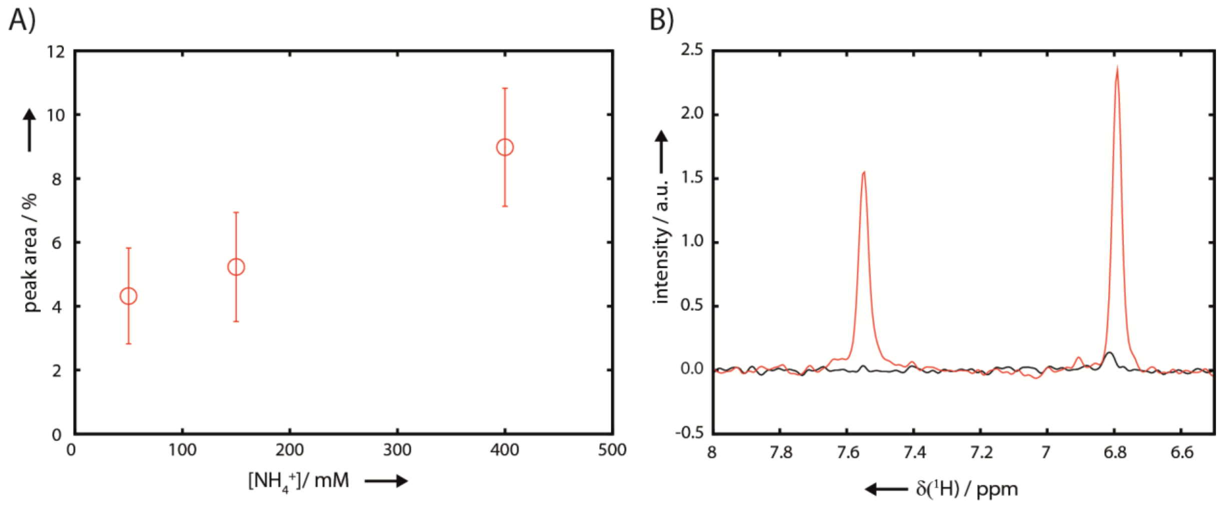

Figure S1: Titration of $^{15}NH_4^+$ and DnaK-ABD in the absence of nucleotide

A) In the absence of nucleotide only a weak signal in ^{15}N-edited 1D proton spectra could be observed. Peak areas at $\delta(^1H)$ of 6.8 ppm with increasing concentrations of $^{15}NH_4Cl$ (50, 150 and 400 mM 15NH_4Cl) are plotted. The peak areas are given in percent relative to the area in the presence of 150 mM $^{15}NH_4Cl$ and ADP-Pi, estimating an upper limit under the experimental conditions. **B)** ^{15}N-edited 1D proton spectra with 400 mM $^{15}NH_4Cl$ (black) and with 150 mM $^{15}NH_4Cl$ and ADP-Pi (red). The concentration of DnaK-ABD was ~ 50 µM, all other buffer conditions were as described in Figure 3 in the main text. It should be noted that the reported peak areas as compared to the nucleotide bound form of DnaK-ABD are only slightly higher than our estimated nucleotide content of the protein preparation (~ 2%, see Materials and Methods) and therefore these data should be interpreted with care. Nonetheless, we can conclude that the nucleotide free form of DnaK-ABD either binds NH_4^+ with extremely weak affinity (K_D > 400 mM) or that the exchange of the bound ammonium ions with the solvent is much more rapid than in the nucleotide bound form.

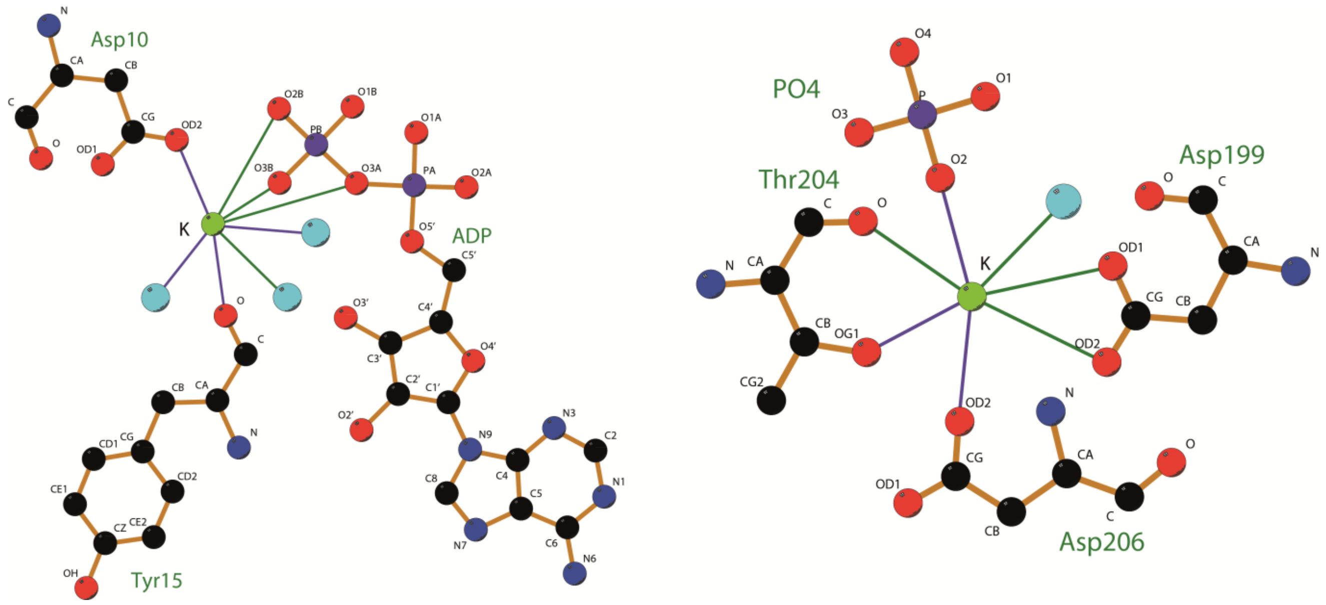

Figure S2: Potassium binding sites of Hsp70-ABD

This figure is modified from one generated using LigPlot+ (as in Figure 1) and the PDB file 1HPM (see Figure 3). In addition to the interactions with the potassium ion generated by the program (purple lines) we also include those described in the original publication (see reference [2], green lines). Oxygen atoms from water molecules are represented in light blue.

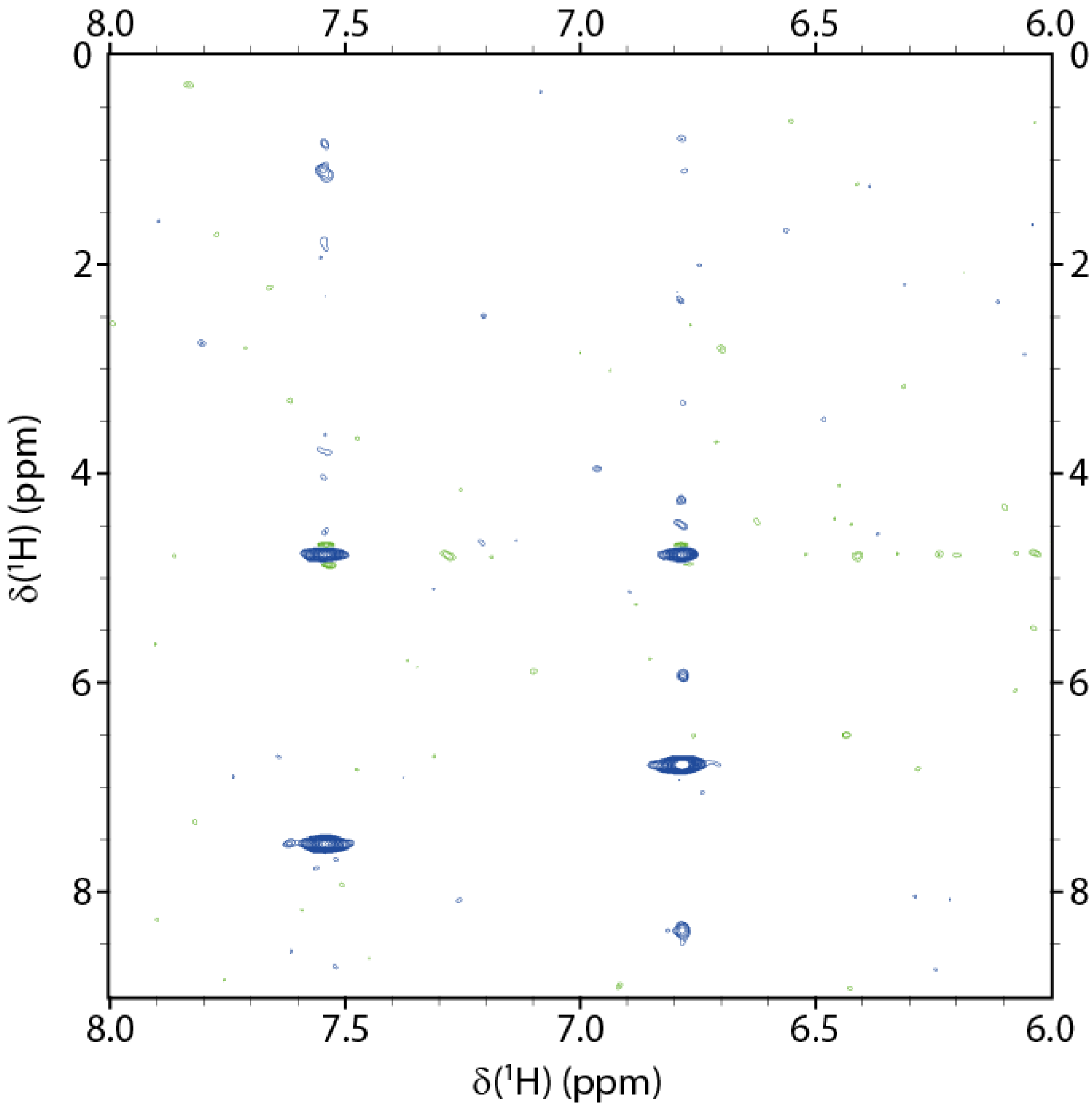

Figure S3: 2D plane of a ^{15}N-edited NOESY experiment with DnaK-ABD.

Buffer conditions and sample are described in Figure 3b). The spectra were recorded on a 500 MHz Bruker Avance III spectrometer as described in Materials and Methods.

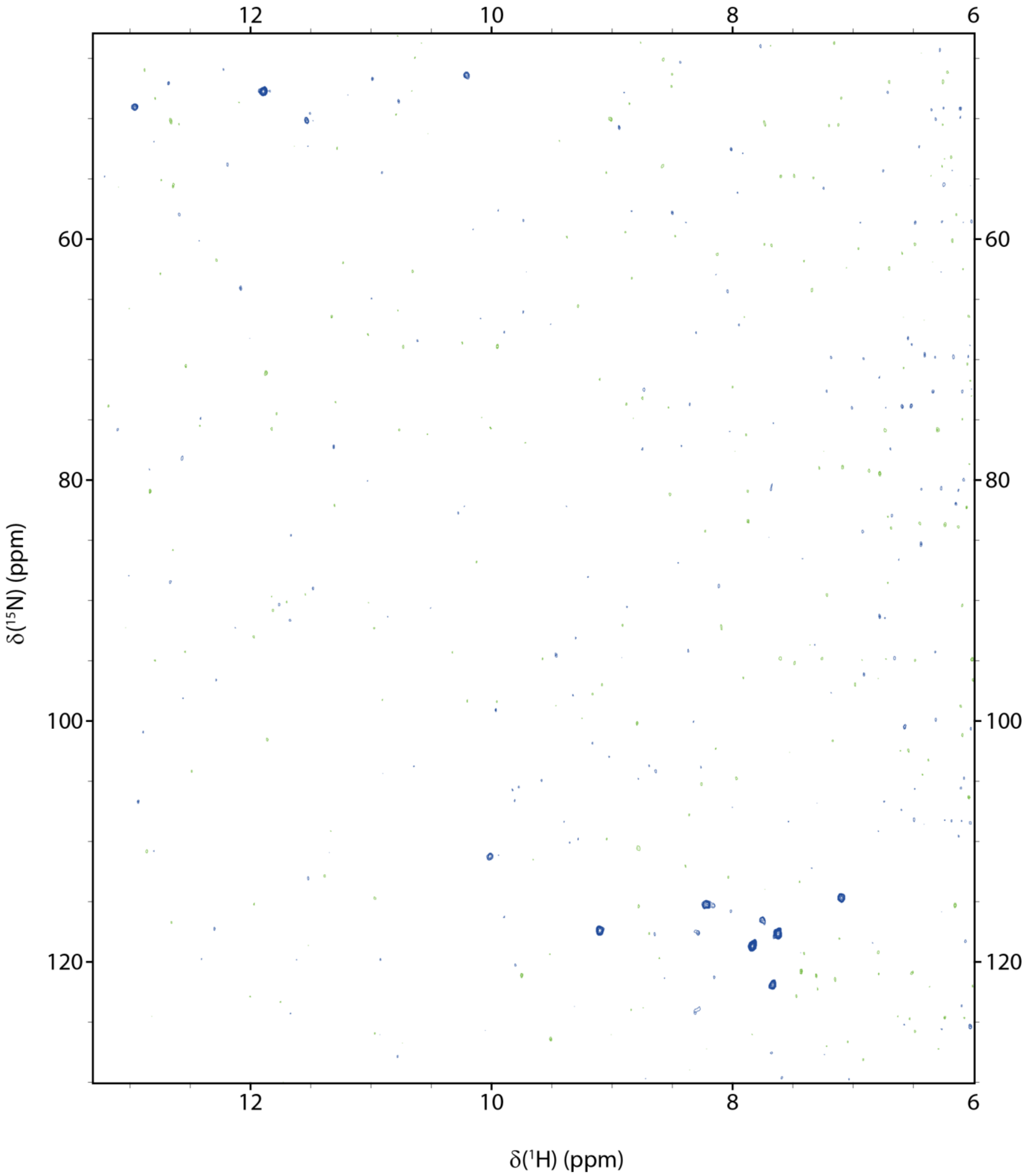

Figure S4: ^{1}H-^{15}N-HSQC spectrum of the ^{1}H/^{15}N His-labeled ^{2}H/^{14}N-HDAC8.

The spectrum is recorded with a wide spectral width in the nitrogen dimension, showing backbone amides (around a ^{15}N chemical shift of 120 ppm) and side-chains (peaks are folded, around 50 ppm) of histidine residues in HDAC8.
